# Rapid Magnetic 3D Printing of Cellular Structures with MCF-7 Cell Inks

**DOI:** 10.34133/2019/9854593

**Published:** 2019-02-04

**Authors:** S. Mishriki, A. R. Abdel Fattah, T. Kammann, R. P. Sahu, F. Geng, I. K. Puri

**Affiliations:** ^1^School of Biomedical Engineering, McMaster University, Hamilton, Ontario, Canada; ^2^Department of Mechanical Engineering, McMaster University, Hamilton, Ontario, Canada; ^3^Faculty of Biological Sciences, Friedrich-Schiller-University Jena, Germany

## Abstract

A contactless label-free method using a diamagnetophoretic ink to rapidly print three-dimensional (3D) scaffold-free multicellular structures is described. The inks consist of MCF-7 cells that are suspended in a culture medium to which a paramagnetic salt, diethylenetriaminepentaacetic acid gadolinium (III) dihydrogen salt hydrate (Gd-DTPA), is added. When a magnetic field is applied, the host fluid containing the paramagnetic salt is attracted towards regions of high magnetic field gradient, displacing the ink towards regions with a low gradient. Using this method, 3D structures are printed on ultra-low attachment (ULA) surfaces. On a tissue culture treated (TCT) surface, a 3D printed spheroid coexists with a two-dimensional (2D) cell monolayer, where the composite is termed as a 2.5D structure. The 3D structures can be magnetically printed within 6 hours in a medium containing 25 mM Gd-DTPA. The influence of the paramagnetic salt on MCF-7 cell viability, cell morphology, and ability of cells to adhere to each other to stabilize the printed structures on both ULA and TCT surfaces is investigated. Gene expressions of hypoxia-inducible factor 1-alpha (*HIF1α*) and vascular endothelial growth factor (*VEGF*) allow comparison of the relative stresses for the printed 3D and 2.5D cell geometries with those for 3D spheroids formed without magnetic assistance. This magnetic printing method can be potentially scaled to a higher throughput to rapidly print cells into 3D heterogeneous cell structures with variable geometries with repeatable dimensions for applications such as tissue engineering and tumour formation for drug discovery.

## 1. Introduction

Two-dimensional (2D) environments, where cells are grown on a tissue culture treated (TCT) surface, have limited clinical relevance since they do not correctly mimic the interactions that influence living cells. In contrast, three-dimensional (3D) models provide more accurate representations of physiologic environments. For 3D cell geometries composed of human carcinoma cells, these interactions involve cell-cell signaling, presence of extracellular matrix (ECM), mechanical cues, hypoxic environments, gene expressions, and drug resistance [1–4]. Examples include multicellular tumours [5, 6], mammospheres formed with mammary cells [7], and tissue spheroids that are embedded in a hydrogel matrix as building blocks to produce larger cell structures [8].

Traditional methods to create 3D spheroid-like cultures require that suspended cells adhere to each other to form nucleation sites that initiate 3D growth. This necessitates the use of nonadhesive surfaces or a liquid-air interface to prevent adherent cells from coalescing and spreading into 2D monolayers. Thus, 3D aggregates are typically grown in a hanging drop setup [9] or on ultra-low attachment (ULA) surfaces [10]. Since culturing techniques limit the ability of some cell lines of forming 3D structures, chemically formulated media, containing reduced amounts of nutrient serum [11] growth factors and additives (including L-glutamine, epidermal growth factor (EGF), basic fibroblast growth factor (bFGF), and reconstituted basement membrane (rBM) [6, 12–14]) are used.

When adherent cells are suspended in medium on flat-bottom ULA plates, multiple 3D masses can be produced in a single well. However, since these masses have nonuniform dimensions, the numbers of spheroids vary from well to well. The hanging drop method circumvents this limitation, allowing cells to aggregate along the liquid-air interface of a cell suspension [15], enabling a structure with uniform dimensions and a specific number of cells for each spheroid. Despite its advantages, the hanging drop method is laborious and time consuming, and also difficult for producing large numbers of spheroids, thereby limiting throughput [16]. Advances in cell manipulation and microscale 3D cell structure formation have incorporated the production of a high-gradient magnetic field in microfluidic devices [17] and for label-free magnetic manipulation [18–22], and into agarose [5] and polydimethylsiloxane (PDMS) [23] microwells.

The addition of diethylenetriaminepentaacetic acid gadolinium (III) dihydrogen salt hydrate (Gd-DTPA) to a cell suspension transforms the medium into a magnetic bioink, where the liquid component of the ink has a higher magnetic susceptibility than the cells contained within it. Therefore, the paramagnetic liquid is more susceptible to a magnetic field than are the suspended diamagnetic cells [18, 24, 25]; i.e., the liquid is preferentially attracted towards the magnetic field while the cells are not. Placing magnets at suitable locations induces ink movement within a vessel, which focuses the suspended cells into 3D structures at locations of lower magnetic field strength, a process called diamagnetophoresis. Since cell patterning through diamagnetophoresis can be controlled and the method foregoes use of nozzles and complicated equipment which can introduce contaminants, it is a convenient technique to rapidly print multicellular spheroids. Potential applications include tissue engineering and drug discovery, allowing the emulation of* in vivo* phenomena in an adjustable* in vitro* environment.

We have previously demonstrated a method to print 3D cellular structures through diamagnetophoresis using a whole blood ink to demonstrate proof of concept [26], and another ink containing a binary mixture of mammalian cell cultures to observe morphological and phenotypic changes in a co-culture [27]. Although it is used as a magnetic resonance imaging (MRI) contrast agent, large Gd-DTPA concentrations can be toxic. Hence, we have evaluated the effect of the paramagnetic salt on human breast cancer cell lines [28]. Cells suspended in a Gd-DTPA medium can also be patterned through diamagnetophoresis on a TCT surface to which cells adhere, forming a relatively small central 3D lump, where a monolayer spreading outward from a central lump is useful for investigating cell migration and fabrication of co-cultures [27]. We call this latter geometry a 2.5D structure since it contains features of both a small 3D spheroid and a 2D monolayer of actively proliferating cells, traditionally observed in transwell assays [29, 30].

We print five types of cell structures with and without diamagnetophoresis using bioinks containing MCF-7 (Michigan Cancer Foundation-7) cells, a human breast cancer cell line. These structures are created to compare diamagnetophoretic printing with traditional methods to characterize the time required to form spheroids, their dimensions and gene expressions. Magnetically assisted bioprinting rapidly prints reproducible 3D and 2.5D structures without compromising the behaviours of the printed structures.

## 2. Results

### 2.1. Effect of Gd-DTPA on Cell Proliferation

**Figure 1:**
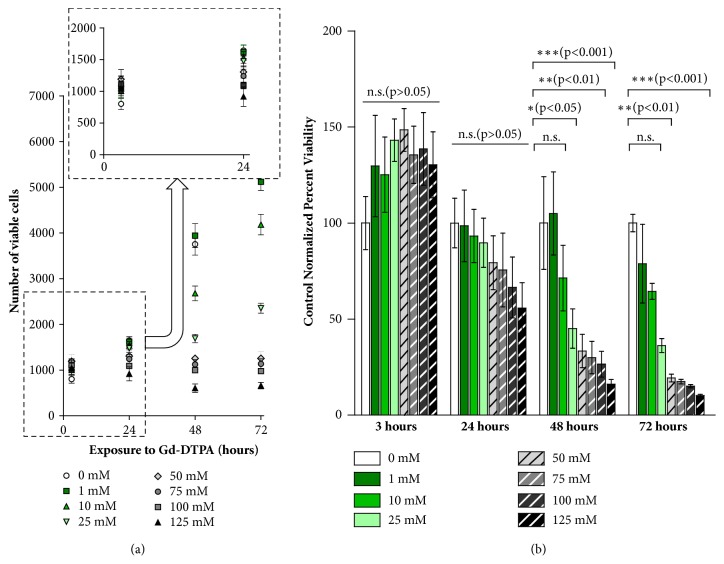
**Effect of Gd-DTPA on cell proliferation**. Approximately, 1000 MCF-7 cells are incubated in 0, 1, 10, 25, 50, 75, 100, and 125 mM Gd-DTPA. Cell proliferation is measured by MTT assay at 3, 24, 48, and 72 hours. The viable cells are (a) quantified by a standard curve (for n=3 analyzed by standard error) and (b) control normalized percent viability using Gd-DTPA free medium (0 mM) using SEM and a two-way ANOVA with Bonferroni posttests to evaluate the relative differences in viability for each concentration of Gd-DTPA. A p < 0.05 is considered to be statistically significant. As the Gd-DTPA concentration increases, cell proliferation is reduced. However, at 24 hours, the effects of Gd-DTPA to cell viability are similar to that of Gd-DTPA free medium. Significant decreases in cell viability are observed for MCF-7 cells in 25 mM and above Gd-DTPA at 48 and 72 hours. Therefore, exposure to Gd-DTPA should be limited to a maximum of 24 hours in order to limit harmful effects of Gd-DTPA on cell proliferation.

The paramagnetic culture medium consists of Gd-DTPA salt dissolved in DMEM supplemented with 10% FBS, as described in Materials and Methods. Since the salt is toxic at high concentrations and prolonged exposures [18, 27, 28, 31], we assess the proliferation of MCF-7 monolayers incubated with 0, 1, 10, 25, 50, 75, 100, and 125 mM Gd-DTPA dissolved in the cell culture medium. For cells exposed to each concentration of Gd-DTPA, an MTT assay quantifies viable cells at 3, 24, 48, and 72 hours.  Figure 1(a) shows that as the exposure time and Gd-DTPA concentration increase, the number of viable cells is diminished. At three hours of exposure to Gd-DTPA, there is an observable increase in cell proliferation, but at 10 mM there is a decrease in cell proliferation. The proliferation increase is explained in part by the increase in the metabolic activity of the cells in presence of Gd-DTPA [28]. Regardless, the effect of Gd-DTPA is indistinguishable from that of the control (0 mM Gd-DTPA) within the first 24 hours of exposure to the salt, as shown in Figure 1(b), which reports the cell viability normalized to that for a Gd-DTPA-free medium for each incubation period. For all concentrations of Gd-DTPA, at 3 and 24 hours of incubation variabilities in the percent normalized viability are insignificant.

### 2.2. Effect of Gd-DTPA on Cell Morphology

**Figure 2:**
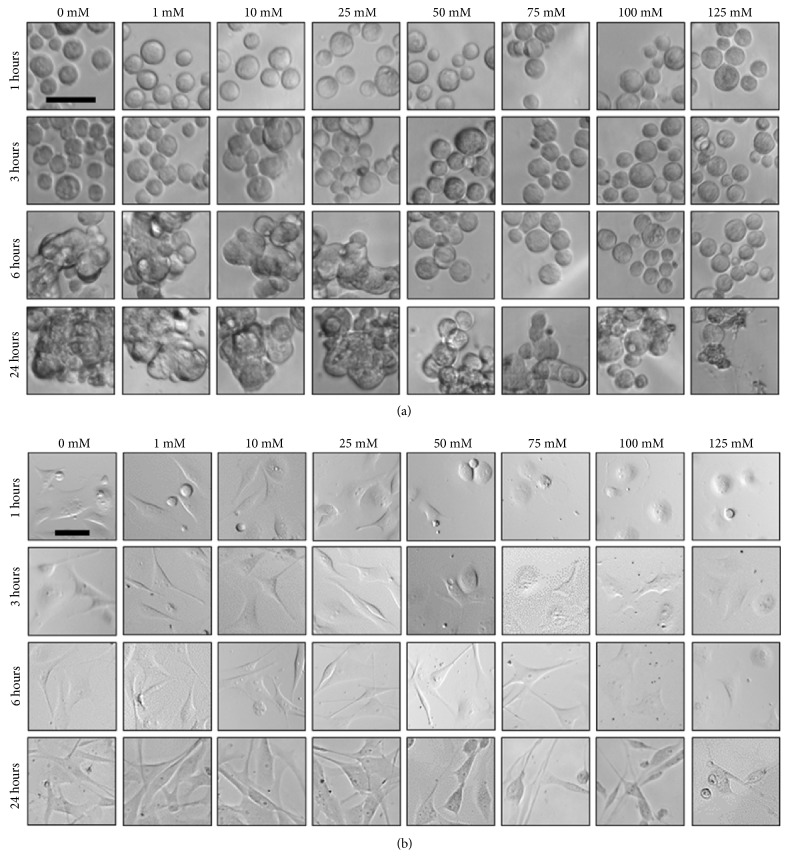
**Effect of Gd-DTPA on cell morphology**. MCF-7 cell morphologies in 0, 1, 10, 25, 50, 75, 100, and 125 mM Gd-DTPA (n=12) within 24 hours when incubated on (a) a ULA plate and (b) TCT plate. Within 6 hours, there are no apparent effects on cell morphology. At concentrations above 25 mM Gd-DTPA at 6 hours, the cell morphologies begins to differ from structures produced with 0 mM Gd-DTPA (control samples) for both ULA and TCT surfaces. In (a), as the concentration of Gd-DTPA ≥ 50 mM, the ability of cells to adhere together diminishes. Cell-cell adhesion is key for formation of a 3D structure. Similarly, in (b) concentrations of ≥ 50 mM limit intercellular attachment within 1-3 hours of exposure to Gd-DTPA. However, at 6 hours, intercellular adhesion overcomes the limiting influence of Gd-DTPA on cell-cell attachment. Therefore, to reduce harmful effects on cell morphology, exposure to Gd-DTPA should be limited to 25 mM for at most 6 hours. Scale bar = 50 *μ*m.

The appropriate incubation period for MCF-7 cells in Gd-DTPA and the influence of a magnetic field for cell patterning are next evaluated.  Figure 2 presents results for MCF-7 cells incubated on ULA and TCT surfaces in the 0, 1, 10, 25, 50, 75, 100, and 125 mM Gd-DTPA solutions. Cell proliferation is observed at 1, 3, 6, and 24 hours. For the ULA plate (Figure 2(a)), there is no difference between cells contained in all concentrations of Gd-DTPA and the Gd-DTPA-free medium (0 mM) at 1 and 3 hours. At 6 hours, cell-cell adhesion observed through a transition from flat to fused structures is only seen for 0-25 mM Gd-DTPA solutions, indicating that higher concentrations of Gd-DTPA impede cell adhesion, which is required to produce 3D spheroids. At 24 hours, adhesion is observed for all Gd-DTPA concentrations. For cells incubated on TCT surfaces (Figure 2(b)), MCF-7 cells display a variety of morphologies as both time and Gd-DTPA concentrations increase, including circular single-cells prior to attachment to the TCT surface, as well as elongated structures after some time, providing evidence of cell-surface adhesion and attachment. Similar to cells on the ULA surface, from 1 to 3 hours Gd-DTPA concentrations of 50 mM and above prevent intercellular adhesion. At 6 hours, intercellular adhesion overcomes the influence of Gd-DTPA that limits cell-cell attachment. Therefore, the concentration of Gd-DTPA for cells on both surfaces is limited to 25 mM to produce either spheroids or healthy monolayers during a maximum exposure of 24 hours.

### 2.3. Effect of Gd-DTPA to Guide 3D and 2.5D Structure Formation

**Figure 3:**
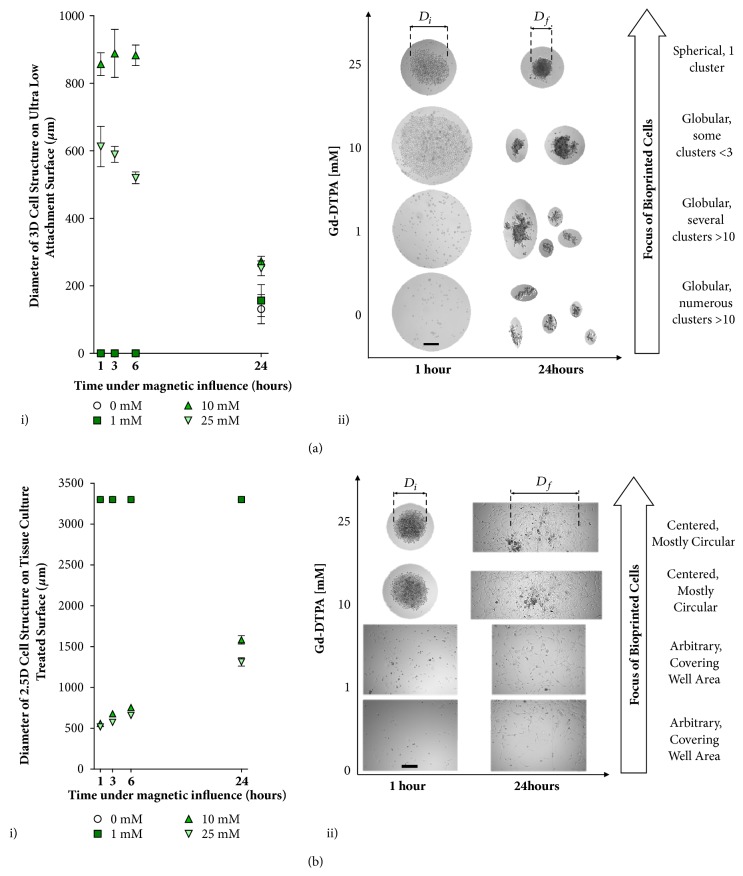
**Effect of Gd-DTPA on diamagnetic cell printing**. Formation of 3D and multidimentional cell structures (2.5D) through diamagnetophoresis on (a) a ULA surface and (b) a TCT surface. Approximately 1000 cells (n=5 analyzed by SD) are incubated in both cases. For (a, i), 0 and 1 mM Gd-DTPA are insufficient to coalesce cells into a 3D structure. At 24 hours, accumulation of numerous globular cluster aggregates is observed. Only 25 and 10 mM Gd-DTPA are able to print cells through diamagnetophoresis. (a, ii) As the concentration of Gd-DTPA decreases from 25 mM, the formation of globular cell clusters increases and their ability to form a single spherical 3D structure is reduced. Only 25 mM is able to produce a single spherical cluster that remained intact until 24 hours. For (b, i), concentrations of 0 and 1 mM are again insufficient to coalesce cells into a 3D structure. The diameters of the cellular structures are equivalent those of their wells since these cells have formed 2D monolayers. Only 10 and 25 mM Gd-DTPA were able to produce a 3D structure; however, for (b, ii) 25 mM Gd-DTPA produced a denser 3D structure. Therefore, 25 mM Gd-DTPA is an appropriate concentration for forming 3D cell structures using diamagnetophoresis. Scale bar = 50 *μ*m.

Figure 3 provides the minimum Gd-DTPA concentration required to coalesce cells together through diamagnetophoretic printing, which is determined by the formation of singular and concentrated 3D and multidimensional (2.5D) cell structures on ULA and TCT surfaces within 24 hours. For inks incubated in 0-25 mM Gd-DTPA and printed through diamagnetophoresis on the ULA surface (Figure 3(a)), while the 10 and 25 mM Gd-DTPA solutions allow 3D spheroids to form, the spheroid diameter* D* decreases significantly for both cases between 6 and 24 hours. Spheroids are unable to form within 6 hours of incubation with 0 and 1 mM solutions (Figure 3(a), i). Use of a 10 mM solution results in the formation of multiple globular clusters and at 24 hours more than ten such clusters are observed (Figure 3(a), ii). For cells magnetically printed on a TCT surface in 0-25 mM Gd-DTPA (Figure 3(b)), again with 10 and 25 mM Gd-DTPA solutions, the diameters of the centered and mostly circular 2.5D cell structures increase over 24 hours, while 0 and 1 mM solutions are unable to direct magnetic assembly (Figure 3(b), ii). The 25 mM solution also produces smaller structures than one with 10 mM (Figure 3(b), i). Therefore, the 25 mM Gd-DTPA solution is used for further experiments with both ULA and TCT surfaces due to its ability to form the desired 3D and 2.5D structures and its limited influence on cell viability and morphology.

### 2.4. Optimization of Incubation Period with Gd-DTPA and Magnetic Field

**Figure 4:**
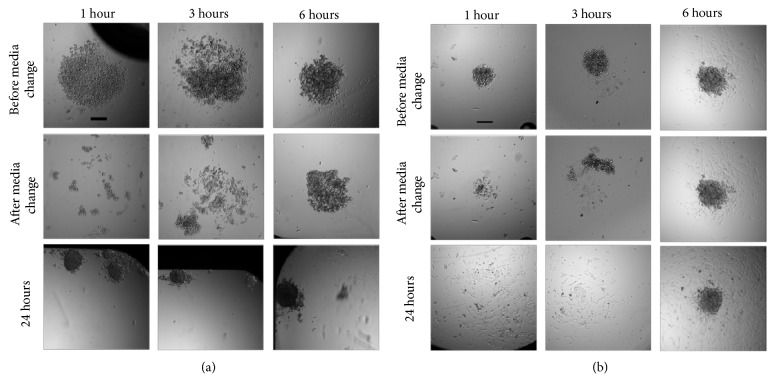
**Incubation period of cells in the presence of external magnetic field**. Cell aggregates following washes with a nonparamagnetic medium after diamagnetic 3D cell printing of (a) 5000 MCF-7 cells on a ULA surface (n=3) and (b) 3000 MCF-7 cells on a TCT surface (n=3) in 25 mM Gd-DTPA for 1, 3, 6, and 24 hours. Incubations periods indicate durations for exposure to the paramagnetic medium and the externally applied magnetic field after which the medium is replaced by 0 mM Gd-DTPA removed to prevent overexposure of Gd-DTPA and the magnetic field. At 1 and 3 hours of incubation, the cells are successfully concentrated to the zones of low magnetic field strength, which are determined by the arrangements of the magnets. However, following medium changes to remove Gd-DTPA, the 3D structures do not maintain their aggregated structures. Only for 6 hours of exposure do the cells remain as a 3D structure following medium changes. Therefore, a minimum of 6 hours is sufficient for producing a single cell structure for cell suspensions in both ULA and TCT surfaces. Scale bar = 200 *μ*m.

The presence of Gd-DTPA is only required to coalesce, or print, the cell suspension, into a single, circular cell structure. After the intended structure has been printed, the medium can be changed to remove the paramagnetic salt. As shown in Figure 3, one hour is sufficient time to print cells into the region of minimal magnetic field strength. However, if the medium is subsequently replaced after 1 or 3 hours of incubation, the intercellular adhesion is insufficient for the spheroid to remain intact and maintain its structural integrity. After 6 hours, the cell-cell adhesion is sufficient to maintain the 3D morphology following a medium change for both ULA (Figure 4(a)) and TCT surfaces (Figure 4(b)). This is consistent with observations of cell morphology from Figure 2, where intercellular adhesion is observed for the 25 mM Gd-DTPA solution after a 6-hour incubation. Hence, in our experiments, in the presence of magnetic field with a 25 mM solution, a 6-hour minimum incubation is maintained to ensure the integrity of a 3D MCF-7 structure following medium change.

### 2.5. Formation and Growth of Spheroids on Various Surfaces

**Figure 5:**
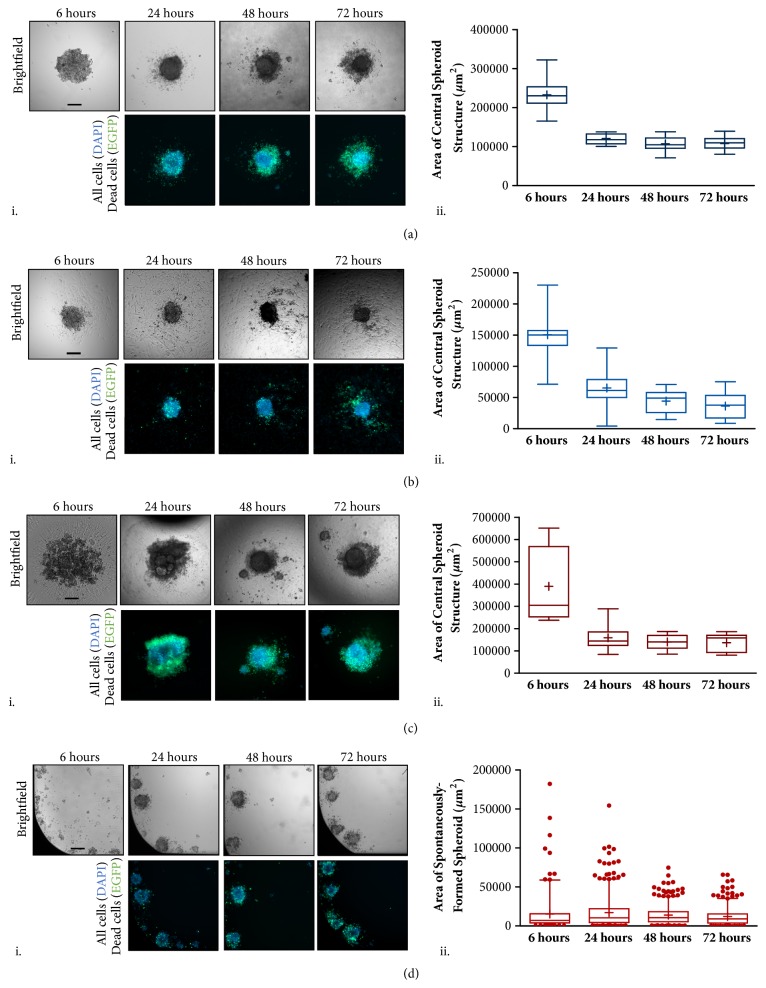
**Growth and viability of cell structures formed by diamagnetophoresis.** Box-and-whisker plots for area measurements of 3D cell structures (n=3) are formed on (a) a flat ULA surface (3D) and (b) a TCT surface (2.5D), as well as 3D structures using (c) round ULA plates that allow the formation of self-assembled spheroids and (d) flat ULA plates to allow the formation of numerous spontaneously formed spheroids per well. Central 3D cell structures were (i) imaged and (ii) measured at 6 hours (following medium changes to remove Gd-DTPA), 24, 48, and 72 hours. Upper and lower whiskers are placed at the 95^th^ and 5^th^ percentile, respectively. Points beyond the whisker ranges are plotted as single dots. At 6 hours, there is a relatively large variation between the forms of the 3D structures. However, at 6 hours, the level of variation between (a) 3D spheroids printed with diamagnetophoresis on a flat ULA surface is much lower than for (c) 3D spheroids printed on a round ULA surface. At 24 hours, the projected areas of both samples are equivalent. Scale bar = 200 *μ*m.

After 6 hours of incubation in the paramagnetic medium in the presence of a magnetic field, the culture medium is changed to remove the Gd-DTPA, as shown in Figure 4. The structures are then observed for an additional 66 hours, i.e., a total of 72 hours. Cell coalescence by the magnetic field initiates intercellular interactions that form the spheroid, but the 3D structures contract due to the dynamic activity of cadherins, a family of Ca^+^- dependent transmembrane proteins involved in epithelial cell anchorage [12, 16, 32]. Live/dead staining is performed for the spheroids at 24, 48, and 72 hours (Figure 5). DAPI (blue) stains all cell nuclei present, while EGFP (green) is specific to dead cells. Overlays of these two images provide references for live (blue) and dead (green) areas. The spheroids, grown for 72 hours, maintain a viable 3D core structure.

A box-and-whisker plot is used to display distribution of the measured dimensions for 3D spheroids formed on various surfaces. The 25^th^ percentile (first quartile, Q1), 50^th^ percentile (median), and 75^th^ percentile (third quartile, Q3) are shown as lines of the box from bottom to top, respectively. The interquartile range (IQR) is the difference between the 25^th^ and 75^th^ percentile for each sample population. Upper and lower whiskers are plotted at the 95^th^ and 5^th^ percentile, respectively. Points beyond the range of the whiskers are plotted as single dots while the mean of each sample is identified by a ‘+' symbol. For 3D spheroids formed diamagnetically in a flat ULA surface (Figure 5(a)), the IQR ranges from 213,481 *μ*m^2^ to 251,519 *μ*m^2^ at 6 hours and reduces to 109,671 *μ*m^2^ to 132,206 *μ*m^2^ at 24 hours. Mean and median values are similar to one another at 6, 24, 48, and 72 hours, indicating a normal distribution of spheroid dimensions. For 2.5D cell structures formed on TCT surfaces (Figure 5(b)), the IQR ranges from 139,854 *μ*m^2^ and 154,979 *μ*m^2^ at 6 hours and 52,544 *μ*m^2^ to 69,210 *μ*m^2^ at 24 hours. Again, the box-and whisker plot is symmetrical and the median is close to the mean value, indicating a normal distribution of the 3D structure. For self-assembled spheroids on a round ULA surface (Figure 5(c)), the IQR ranges from 265,943 *μ*m^2^ to 566,390 *μ*m^2^ at 6 hours and contracts to 124,773 *μ*m^2^ to 168,306 *μ*m^2^ at 24 hours. Since a symmetrical box-and-whisker plot indicates a normal distribution of data, a nonnormal distribution is observed at 6 hours due to the irregularities of spheroid dimensions. Although the final IQR for dimensions of diamagnetically formed spheroids on a flat ULA surface and a round-bottom ULA surface structures at 72 hours are similar (97,713 *μ*m^2^ to 118,817 *μ*m^2^ and 98,115 *μ*m^2^ to 167,998 *μ*m^2^, respectively), the sizes of the self-assembled spheroids printed on round-bottom ULA surfaces have a larger distribution in comparison to the diamagnetically formed spheroids that are printed on flat ULA surfaces. When a cell suspension is placed on a flat-bottom ULA surface, numerous spheroids are formed in each well, where it is not possible to control either their numbers or dimensions, and hence these are termed as* spontaneously-formed* spheroids. The sizes of the spontaneously-formed spheroids in different wells remain virtually unchanged between 6 and 72 hours (Figure 5(d)), but the size distributions however decrease. [Supplementary-material supplementary-material-1] shows the mean projected area measurements of 3D spheroids grown on various surfaces and their respective circularity values, which is summarized in [Supplementary-material supplementary-material-1]. The circularity of the projected areas of magnetically formed spheroids approaches unity, indicating a perfect circle in comparison to that for self-assembled spheroids and spontaneously-formed spheroids which are more irregular.

### 2.6. Gene Expression

Gene analysis is performed with real-time quantitative polymerase chain reaction (qPCR) to assess the relative stresses for the magnetically printed 3D and 2.5D structures and compared with those for 3D structures produced without a magnetic field and Gd-DTPA. Four samples, (1) 3D diamagnetically printed structures on a flat ULA surface, (2) 2.5D diamagnetically printed structures on a flat TCT surface, (3) 3D self-assembled spheroids on a round-bottom ULA surface, and (4) spontaneously- formed spheroids are normalized by their fold-change gene expressions relative to control glyceraldehyde 3-phosphate dehydrogenase (*GAPDH*) to 2D cultures grown on a flat TCT surface.

Hypoxia-inducible factor 1-alpha (*HIF1α*) is a general marker of stress for stress caused by hypoxia, or lack of oxygen. Above a critical diameter of roughly 500 *μ*m, aqueous nutrients in the microenvironment and oxygen are unable to penetrate a 3D MCF-7 structure, leading to hypoxic regions and a necrotic core [2, 33, 34].* HIF1α* is typically overexpressed in 3D structures but can also appear in 2D monolayers of highly proliferative cells [35]. Vascular endothelial growth factor (*VEGF*) is an angiogenic factor that is a classic marker for hypoxic stress shown to be correlated with chemoresistance typically observed in 3D cell structures [5]. 3D tumour structures overexpress* VEGF* to induce tumour vascularization, which is characteristic of tumours* in vivo*.

**Figure 6:**
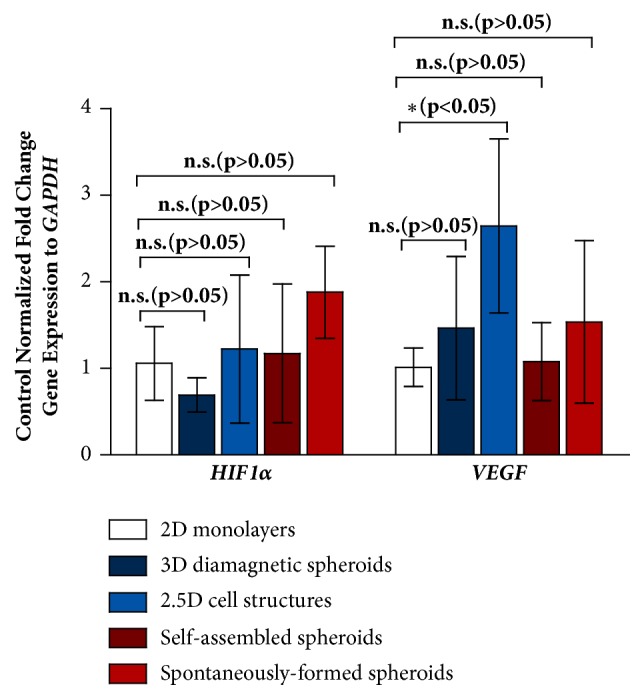
**Control normalized fold change gene expression to** **G** **A** **P** **D** **H**. Expression of **HIF**1*α* is not significant for 3D and 2.5D cell structures in comparison to the normalized expression in 2D monolayers. Expression of **VEGF** is significant only for 2.5D cell structures.

Primer sequences used in the qPCR analysis are given in [Supplementary-material supplementary-material-1]. The gene expressions for* HIF1α* and* VEGF* are shown in Figure 6. For* HIF1α* gene expression, all cell structures are not observed to be under significant hypoxic stress in comparison to the normalized expression in 2D monolayers. This is attributed to the small dimensions of the 3D cell structures, which permit sufficient oxygen diffusion and prevent the formation of a hypoxic region. In comparison to the normalized expression in 2D monolayers, no significant changes are observed for* VEGF* expression in 3D diamagnetic spheroids, self-assembled spheroids, and spontaneously-formed spheroids. However, 2.5D cell structures overexpress* VEGF* in comparison to 2D monolayers, which can be attributed to the different morphologies observed in the 2.5D cell landscape [27], that suggest the presence of unique gene expressions found in human breast cancers [36].

## 3. Discussion

We describe a rapid method to print multidimensional tumours with bioinks containing MCF-7 cells. The resulting cell structures include two types formed through diamagnetophoresis, i.e., 3D spheroids on (1) a ULA plate and (2) a 2.5D lump and spreading monolayer on a TCT surface, as well as three structures formed without magnetic assistance, i.e., (3) 3D spheroids on a round-bottom ULA plate (self-assembled spheroids) (4) 3D spheroids produced on a flat bottom ULA plate (spontaneously-formed spheroids), the counterpart to Case 1, and (5) 2D monolayers grown on a TCT surface, the counterpart to Case 2. The magnetically printed tumours have reproducible geometries that can be varied by adjusting the strength and orientation of the magnetic field external to the wells. These tumours can be printed within 6 hours, more rapidly than self-assembled tumours can be formed on round-bottom ULA plates. Varying the number of cells changes the spheroid size. Gene expression analysis of printed and self-assembled spheroids, i.e., cells that coalesce without being magnetically manipulated, is indistinguishable, proving that the method is capable of producing viable cell structures in 3D geometries.

MCF-7 cells are among the most-researched human breast cancer cell lines.[37] MCF-7 cells have been reported in numerous studies for the formation of 3D spheroids and mammospheres, making them a suitable candidate for the investigation of magnetic printing of 3D cell structures through diamagnetophoresis. An* in vitro* 3D cell structure composed of human adenocarcinoma cells is relevant for studies of drug response and metastasis. This cancer drug model can be complexed through the addition of stromal cells [38] to mimic the microenvironment of the tumour site, thereby establishing a platform which can provide greater clinical relevance than a monotypic cell landscape.

This method is feasible for other cell lines. However, careful consideration of the total exposure to Gd-DTPA (including concentration and incubation time) must be made to optimize the formation of 3D cell structures. The magnetic force on a cell, **F**_m_[26], were

(1) $$\begin{array}{c} F_{m}=\left(\;\frac{\left(\chi_{c}-\chi_{m}\right)}{2\mu_{0}}\;\right)V_{c}\nabla {\left|\;B\;\right|}^{2} \end{array}$$

where *χ*_c_ and *χ*_m_ are the magnetic susceptibilities of the cell and of the fluid culture media, respectively, *μ*_0_ is the permeability of free space, *V*_c_ is the cell volume, and ∇|**B**|^2^ is the magnetic field gradient. For cells of the same size and magnetic susceptibility, their movement of within the medium behaves similarly when the magnetic field gradient is maintained. Therefore, the incubation time required to form a stable 3D cell structure depends on the excretion of extracellular proteins to provide structural support and maintain the integrity of the printed cell structure. Using this method, it is expected that the use of other cell lines will demonstrate accelerated 3D formation, as observed with MCF-7, due to the decreased proximity of cells to one another, which increases the cell-cell contacts to produce ECM proteins.

This system offers a label-free, scaffoldless approach to printing 3D cell structures* in vitro*. However, this system may be limited by the ability to generate sufficient convection within the cell suspension for low cell numbers, using the current setup, to achieve a 3D cell geometry. To circumvent this, the well volume can be decreased to promote spatial displacement of the cells from the movement of the paramagnetic media towards high magnetic field force. In addition, the number of occupied wells in each plate is limited by the size of the magnets underneath each well which is used to form a single spheroid. Therefore, the use of smaller magnets can be used, only in the case that they are able to induce sufficient magnetic force on the cell suspension.

This technique for magnetic assembly offers a solution to forming reproducibly sized 3D MCF-7 cell structures more rapidly (within 6 hours), compared to those formed by the use of agarose microwells, which require a 24-hour sedimentation period to prevent breaking of the 3D cell structure [39]. In addition, this scaffoldless method does not require additives in culture media, as is seen in hanging drop [40] and liquid overlay [41] techniques. This intentional bioprinting method of cell coalescence has applications to tissue engineering, drug testing, and cell-on-chip devices, thus providing a means to miniaturize simple* in vivo* cell structures for physiologically relevant cell models.

## 4. Materials and Methods

### 4.1. Materials and Reagents

Dulbecco Modified Eagle's medium (DMEM, Life Technologies, cat# 12800-082) containing 10% fetal bovine serum (FBS, cat# 12484028) was used as the cell culture medium. Diethylenetriaminepentaacetic acid gadolinium (III) dihydrogen salt hydrate (97%, gadopentatic acid, Gd-DTPA) was purchased from Sigma Aldrich, Canada (cat# 381667). MTT (3-(4,5-dimethylthiazol-2-yl)-2,5-diphenyltetrazolium bromide) reagent was purchased from Invitrogen, Canada (cat# M6494). For cell culture maintenance phosphate buffered saline (PBS, cat# 10010023) and Trypsin-EDTA (0.25%), phenol red (cat# 25200056) was purchased from Life Technologies, Canada. Dimethyl sulfoxide (DMSO) was purchased from Sigma Aldrich, Canada (cat# D4540). Various cell culture plates were utilized for the preparation of 2D and 3D samples (Corning, Canada): 6-well TCT plates for 2D monolayers (ref# 353046); 384-well ULA plates (cat# 3837) with and without an external magnetic field for the formation of 3D spheroids and spontaneously-formed spheroids, respectively; 96-well TCT plates (ref# 4680) with an external magnetic field for the formation of 2.5D spheroids; and 384-well spheroid microplates (ref# 4516) were used for the formation of self-assembled spheroids. The NdFeB grade N52 magnets were purchased from Zigmyster Magnets, with dimensions of 3.175×3.175×3.175 mm. Other reagents used include sodium hydroxide (NaOH) (Alfa Aesar, cas# A16037), 2-mercaptoethanol (99%, Sigma Aldrich, Canada), and ethanol (Commercial Alcohols, Canada, cat# P016EAAN).

### 4.2. Characterization Methods and Instruments

ReadyProbes™ Cell Viability Imaging Kit, Blue/Green (Invitrogen, Canada, cat# R37609), was used to stain spheroid samples for fluorescence imaging. Optical brightfield and fluorescence imaging (enhanced green fluorescent protein (EGFP), and 4′,6-diamidino-2-phenylindole (DAPI) stains) were performed using a Carl Zeiss Axio Observer.Z1 microscope. Excitation and emission wavelengths of 395/509 and 358/461 were used for EGFP and DAPI, respectively. The Tecan Infinite M200 Pro was used for MTT absorbance readings. Size measurements of the central assembled 3D structures were evaluated using ImageJ. The Dual 48/48W Fast and CFX96 thermal cyclers (BioRad, United States) were used for reverse transcriptase and real-time qPCR, respectively.

### 4.3. Synthesis of Paramagnetic Gd-DTPA Medium

A stock solution of 150 mM of Gd-DTPA was prepared by mixing 5.47 g of Gd-DTPA in 50 mL of culture medium and adjusted by adding 17 mL of NaOH to reach an isotonic pH of approximately 7.4±0.2. Contents were constantly mixed on a stir plate as Gd-DTPA and NaOH were added.* Note*. readjusting pH of the culture medium following the addition of Gd-DTPA must be done quickly in order to preserve the medium's nutritional contents. The paramagnetic medium was then sterilized using a 0.22 *μ*m filter.

### 4.4. MTT Assay Analysis

MCF-7 cells were trypsinized from a culture plate and allowed to form monolayers containing 1000 cells per well were incubated in 100 *μ*L 0, 1, 10, 25, 50, 75, 100, and 125 mM Gd-DTPA medium in 96-well TCT plates at standard conditions (37°C, 5% CO_2_ in a humidified incubator). At 3, 24, 48, and 72 hours, cell proliferation was analyzed by the MTT assay. MTT reagent was diluted in sterile PBS to achieve a final concentration of 5 mg/mL. At each incubation time i.e. 3, 24, 48, and 72 hours, a standard curve was prepared to quantify the unknown number of viable cells present in each incubated sample of Gd-DTPA. 10 *μ*L MTT reagent was added to each well and left to incubate at standard conditions for 3 hours. Following incubation with MTT reagent, all but 25 *μ*L sample volume was removed from each well. 50 *μ*L DMSO was then added to each well, and the plate was left to incubate at standard conditions for 10 minutes. The plate was shaken for 5 seconds, and the absorbance read at 570 nm. For each exposure time, three biological triplicates (n=3) with six technical triplicates for each Gd-DTPA concentration were performed.

### 4.5. Morphology Analysis

To observe effect of Gd-DTPA on cell morphology, 1000 MCF-7 cells were incubated under standard conditions in 0, 1, 10, 25, 50, 75, 100, and 125 mM Gd-DTPA culture medium in 384-well ULA and 96-well TCTplates. Images were taken at 1, 3, 6, and 24 hours at 40× magnification using brightfield microscopy.

### 4.6. Preparation of 3D and 2.5D Geometries Formed by Diamagnetophoresis

MCF-7 cells were plated in 384-well ULA and 96-well TCT plates for the preparation of 3D spheroids and 2.5D cell structures, respectively, and incubated under standard conditions. Cells were suspended in paramagnetic medium under the influence of a magnetic field by arranging a quartet of magnets in N-S-N-S orientation centered directly underneath each well. After cells in the 3D and 2.5D samples have reached maximum accumulation into a single zone of zero magnetic field strength, medium changes were performed in each well using 0 mM Gd-DTPA to dilute concentration of Gd-DTPA to below 1 mM Gd-DTPA. 3D and 2.5D geometries were formed in 80 *μ*L of medium per well.

### 4.7. Effect of Gd-DTPA to Form Spheroids

1000 MCF-7 cells were plated in 0, 1, 10, and 25 mM Gd-DTPA to form 3D spheroids and 2.5D cell structures, respetively, as described above. Images were taken at 1, 3, 6, and 24 hours at 5× magnification using brightfield microscopy. ImageJ was used to analyze changes in spheroid size. A sample size of n=5 was used for each concentration of Gd-DTPA for both 3D and 2.5D samples.

### 4.8. Effect of Incubation Period with Gd-DTPA and Magnetic Field for Formation of 3D and 2.5D Structures

5000 and 3000 MCF-7 cells were plated in 25 mM Gd-DTPA to form 3D spheroids and 2.5D cell structures, respectively, as described above. At 1, 3, 6, and 24 hours, magnets were removed from underneath the samples, and the paramagnetic medium was replaced. Images were taken at 5× magnification. A sample size of n=5 was used for both 3D and 2.5D samples in biological duplicates.

### 4.9. Preparation of 2D, 3D, and 2.5D Samples for Size and Gene Expression Analysis

2D monolayer samples were prepared by seeding 100,000 MCF-7 cells in 0 mM Gd-DTPA (sample control) in a 6-well TCT plate, and incubated under standard conditions. 3D spheroids and 2.5D cell structures formed through diamagnetophoresis were formed by seeding 5000 and 3000 MCF-7 cells in 25 mM Gd-DTPA, respectively, as described above. At 6 hours, magnets were removed from underneath the samples, and the paramagnetic medium was replaced. For self-assembled spheroids, 5000 cells were seeded into a 384-well spheroid microwell plate containing 80 *μ*L of 0 mM Gd-DTPA medium. For spontaneously-formed spheroids, 5000 cells were seeded in 384-well ULA plate using 80 *μ*L of 0 mM Gd-DTPA medium. Images of 3D and 2.5D samples were taken at 6, 24, 48, and 72 hours of incubation under standard conditions at 5× magnification. ImageJ was used to analyze changes in spheroid size.

### 4.10. qPCR Measurements for Gene Expression Analysis

Single-stranded ribonucleic acid (RNA) was extracted from all 2D, 3D, and 2.5D samples after 72 hours of incubation under standard conditions using E.N.Z.A.® HP Total RNA Kit (Omega Bio-Tek, United States, cat# R6812) according to the manufacturer's specifications. 1 *μ*g of RNA was reverse transcribed using SuperScript™ IV VILO Master Mix (Invitrogen, Canada, cat# 11756050) for the synthesis of complementary deoxyribonucleic acid (cDNA). The cDNA product was then used for quantitative polymerase chain reaction (qPCR) analysis using PowerUp™ SYBR™ Green Master Mix (Invitrogen, Canada, cat# A25918). PCR protocol was performed as follows: 50.0°C for 2 minutes, 95.0°C for 2 minutes, and 40 cycles of 95.0°C for 15 seconds, 60.0°C for 15 seconds, and 72.0°C for 1 minute. The plate was read at the end of each cycle. Primer sequences were purchased from IDT (Canada). The gene expression of each sample (relative to the expression levels of 2D monolayer samples) was calculated using the delta-delta (∆∆) cycle threshold (C_T_), 2^(-∆∆CT)^[42], method as follows:

(2) $$\begin{array}{c} \text{Fold change gene expression}=2^{\left(\text{-}∆∆CT\right)} \end{array}$$

where ∆∆C_T_ = [(*HIF1α*/*VEGF*C_T_ -* GAPDH*C_T_) of 2.5D/3D cell structures] - [(*HIF1α*/*VEGF*C_T_ -* GAPDH*C_T_) of 2D monolayer].

### 4.11. Statistical Analysis

MTT semiquantitative results were analyzed by standard error (n=3). Statistical analysis on the control normalized percent viability was performed using standard error of the mean (SEM) and a two-way analysis of variance (ANOVA) with Bonferroni posttests to compare replicate means to Gd-DTPA free media values. Morphology analysis was performed using n=12 for each concentration of Gd-DTPA. The effect of Gd-DTPA to coalesce cells was performed using n=5 for each concentration of Gd-DTPA and results were analyzed by standard deviation (SD). Preparation of 2D, 3D, and 2.5D geometries for gene expression analysis was performed by preparing biological triplicates (n=3) of each sample. 3D and 2.5D samples contained a minimum of 45 technical replicates for each biological triplicate. Statistical analysis was performed using SD and a two-way ANOVA with Bonferroni posttests to compare triplicate means to 2D monolayers. 5 samples from each biological triplicate of prepared 3D and 2.5D samples for gene expression analysis were used for size analysis. All statistical analysis was performed using GraphPad Prism software with a 95% confidence interval.

## Supplementary Materials

Supplementary Materials
** Figure S1. Area of 3D spheroids on various surfaces analyzed by SD.**  (**A**) For magnetically printed spheroids on flat ULA surfaces, their central structures have initial areas of approximately 232,984 ± 71,040 *μ*m^2^ at 6 hours, which contract to 120,760 ± 12,821 *μ*m^2^ at 24 hours. (**B**) For similarly printed 2.5D structures on TCT surfaces, the 3D central structures have initial areas of approximately 150,785 ± 39,585 *μ*m^2^ at 6 hours, which contract to 65,311 ± 30,753 *μ*m^2^ at 24 hours. (**C**) When 3D spheroids are self-assembled on round-bottom ULA plates, the central structures have initial areas of approximately 389,800 ± 161,590 *μ*m^2^ at 6 hours, which also contract to 158,702 ± 55,702 *μ*m^2^ at 24 hours. At 48 and 72 hours, the projected areas of the printed spheroids remain unchanged. (**D**) For spontaneously formed spheroids, the area distribution is significantly skewed at 6 and 24 hours. The dimensions again remain similar at 48 and 72 hours.** Table S1.** Area and circularity measurements of 3D structures up to 72 hours.** Table S2.** Primer sequences used for PCR analysis.
