# Supplementary material for: Rapid Magnetic 3D Printing of Cellular Structures with MCF-7 Cell Inks
Source: Research (Wash D C). 2019 Feb 4;2019:9854593. doi: 10.34133/2019/9854593 (PMC6750075; doi:10.34133/2019/9854593)
Supplement: Supplementary Materials — Figure S1. Area of 3D spheroids on various surfaces analyzed by SD. (A) For magnetically printed spheroids on flat ULA surfaces, their central structures have initial areas of approximately 232,984 ± 71,040 μm2 at 6 hours, which contract to 120,760 ± 12,821 μm2 at 24 hours. (B) For similarly printed 2.5D structures on TCT surfaces, the 3D central structures have initial areas of approximately 150,785 ± 39,585 μm2 at 6 hours, which contract to 65,311 ± 30,753 μm2 at 24 hours. (C) When 3D spheroids are self-assembled on round-bottom ULA plates, the central structures have initial areas of approximately 389,800 ± 161,590 μm2 at 6 hours, which also contract to 158,702 ± 55,702 μm2 at 24 hours. At 48 and 72 hours, the projected areas of the printed spheroids remain unchanged. (D) For spontaneously formed spheroids, the area distribution is significantly skewed at 6 and 24 hours. The dimensions again remain similar at 48 and 72 hours. Table S1. Area and circularity measurements of 3D structures up to 72 hours. Table S2. Primer sequences used for PCR analysis. [file 9854593.f1.zip › Table S1 and S2.docx]

**Table S1.** Area and circularity measurements of 3D structures up to 72 hours.

|  | **3D Cell Structure** | **Area of central spheroid structure (mean** ± **SD μm^2^)** | **Circularity**  **(mean** ± **SD)** |
| --- | --- | --- | --- |
| **6 hours** | 3D diamagnetic spheroids | 232,984 ± 71,040 | 0.4585 ± 0.1459 |
|  | 2.5D cell structures | 157,785 ± 39,585 | 0.4311 ± 0.0880 |
|  | Self-assembled spheroids | 389,800 ± 161,590 | 0.3283 ± 0.1318 |
|  | Spontaneously-formed spheroids | 15,289 ± 23,544 | 0.5048 ± 0.1703 |
| **24 hours** | 3D diamagnetic spheroids | 120,760 ± 12,821 | 0.7651 ± 0.0843 |
|  | 2.5D cell structures | 65,311 ± 30,753 | 0.7471 ± 0.1187 |
|  | Self-assembled spheroids | 158,702 ± 55,702 | 0.6656 ± 0.1572 |
|  | Spontaneously-formed spheroids | 17,063 ± 19,324 | 0.6477 ± 0.1444 |
| **48 hours** | 3D diamagnetic spheroids | 107,184 ± 17,736 | 0.8636 ± 0.0846 |
|  | 2.5D cell structures | 44,294 ± 18,399 | 0.8933 ± 0.0349 |
|  | Self-assembled spheroids | 140,220 ± 31,474 | 0.7191 ± 0.1319 |
|  | Spontaneously-formed spheroids | 13,922 ± 11,653 | 0.7388 ± 0.1176 |
| **72 hours** | 3D diamagnetic spheroids | 107,726 ± 15,894 | 0.8774 ± 0.0583 |
|  | 2.5D cell structures | 36,378 ± 20,777 | 0.9072 ± 0.0249 |
|  | Self-assembled spheroids | 136,603 ± 39,323 | 0.7907 ± 0.0747 |
|  | Spontaneously-formed spheroids | 12,108 ± 11,249 | 0.7922 ± 0.1053 |

**Table S2.** Primer sequences used for PCR analysis.

| **Gene** | **Forward Sequence (5’-3’)** | **Reverse Sequence (5’-3’)** |
| --- | --- | --- |
| *HIF1α* | ATTTTGGCAGCAACGACACA | TGGGTGAGGGGAGCATTACA |
| *VEGF* | TCAAGCCATCCTGTGTGCC | CTTGGTGAGGTTTGATCCGC |
| *GAPDH* | ATCAAGAAGGTGGTGAAGCAGG | GCGTCAAAGGTGGAGGAGTG |
